# Modelling foraging movements of diving predators: a theoretical study exploring the effect of heterogeneous landscapes on foraging efficiency

**DOI:** 10.7717/peerj.544

**Published:** 2014-09-11

**Authors:** Marianna Chimienti, Kamil A. Bartoń, Beth E. Scott, Justin M.J. Travis

**Affiliations:** School of Biological Sciences, University of Aberdeen, Aberdeen, UK

**Keywords:** Animal movements, Heterogeneous landscapes, Foraging efficiency, Modelling, Marine renewable energies, Impacts

## Abstract

Foraging in the marine environment presents particular challenges for air-breathing predators. Information about prey capture rates, the strategies that diving predators use to maximise prey encounter rates and foraging success are still largely unknown and difficult to observe. As well, with the growing awareness of potential climate change impacts and the increasing interest in the development of renewable sources it is unknown how the foraging activity of diving predators such as seabirds will respond to both the presence of underwater structures and the potential corresponding changes in prey distributions. Motivated by this issue we developed a theoretical model to gain general understanding of how the foraging efficiency of diving predators may vary according to landscape structure and foraging strategy. Our theoretical model highlights that animal movements, intervals between prey capture and foraging efficiency are likely to critically depend on the distribution of the prey resource and the size and distribution of introduced underwater structures. For multiple prey loaders, changes in prey distribution affected the searching time necessary to catch a set amount of prey which in turn affected the foraging efficiency. The spatial aggregation of prey around small devices (∼ 9 × 9 m) created a valuable habitat for a successful foraging activity resulting in shorter intervals between prey captures and higher foraging efficiency. The presence of large devices (∼ 24 × 24 m) however represented an obstacle for predator movement, thus increasing the intervals between prey captures. In contrast, for single prey loaders the introduction of spatial aggregation of the resources did not represent an advantage suggesting that their foraging efficiency is more strongly affected by other factors such as the timing to find the first prey item which was found to occur faster in the presence of large devices. The development of this theoretical model represents a useful starting point to understand the energetic reasons for a range of potential predator responses to spatial heterogeneity and environmental uncertainties in terms of search behaviour and predator–prey interactions. We highlight future directions that integrated empirical and modelling studies should take to improve our ability to predict how diving predators will be impacted by the deployment of manmade structures in the marine environment.

## Introduction

Foraging theory is well-developed and has importance for applied ecological problems with examples including the management of large herbivores (Belovsky, 1991), the effectiveness of biological agents in controlling pest populations (Railsback & Johnson, 2011), and most recently in the development of strategies for mitigating human-wildlife conflicts (Baruch-Mordo et al., 2012). There is currently substantial interest in the foraging behaviour of diving marine predators especially in the context of how this may be influenced by the deployment of marine renewable devices. In this contribution we develop a strategic model to represent, in a highly abstracted way, the foraging behaviour of diving seabirds in environments that can include changes to habitat heterogeneity. Our goal in this paper is to provide some initial theory that provides some general predictions and that can motivate the collection of the types of data that can subsequently be used to develop more refined models that can ultimately yield quantitative, predictive tools that can be used in management.

The early theoretical foraging models assumed that animals make decisions according to optimal decision rules (MacArthur & Piankar, 1966; Schoener, 1971). Animals had full knowledge about resource distribution and could maximize their foraging efficiency (Charnov, 1976). Later models accounted for imperfect information and assumed random and unpredictable resource environments (Bovet & Benhamou, 1991; Bartumeus et al., 2005; Conradt et al., 2003). Foragers used the information gained while foraging to estimate patch quality or prey density detecting prey items only from a limited distance. The information received determined how long a forager will stay in a particular patch (Green, 1980; Olsson & Holmgren, 1998; Valone, 1992).

Movements are key elements in the study of behavioural ecology as they define the interactions between individuals and their environment and their pattern may depend on the distribution of the resources and on other species in the landscape (McKenzie, Lewisa & Merrill, 2009). An animal’s movement decisions can be made both using information about the surrounding habitat within the animal’s perceptual range (Palmer, Coulon & Travis, 2011) and spatial memory (Barraquand, Inchausti & Bretagnolle, 2009). Energetic costs associated with movement in heterogeneous landscapes have also been taken into account (Faustino et al., 2007; Palmer, Coulon & Travis, 2011). Depending on their diet, prey distribution and abundance, predators can show different kind of movements and search tactics ranging from Brownian motions, through correlated random walks, to Lévy walks (Hays, Metcalfe & Walne, 2004; Codling, Plank & Benhamou, 2008; Sims et al., 2008; Humphries et al., 2010). The combination of animal morphology and physiology aspects, characteristics of their food and landscape determine the movement of foraging animals (Lima & Zollner, 1996; Morales & Ellner, 2002; McKenzie et al., 2012). Animal movements have often been modelled using correlated random walks, in which orientation of successive steps is correlated, resulting in directional persistence. This leads to a local directional bias, meaning that each step tends to point to the same direction as the previous one (Byers, 2001; Codling, Plank & Benhamou, 2008). Studies on animal movement patterns provide a basis for understanding their foraging decisions. Movements can be influenced by the patchy configuration of food and can change in response of habitat heterogeneity, and habitat changes across landscape boundaries (Crist et al., 1992; Giuggioli & Bartumeus, 2010; Bartoń & Hovestadt, 2013).

The foraging ecology of top marine predators has been the subject of intensive research in the past decades. Thanks to the recent development of miniaturised data-loggers and survey-based studies, types of movements, important feeding areas and the type of prey that predators are targeting have been identified (Block et al., 2011; Camphuysen et al., 2012). However, information about prey capture rates, the strategies that predators use to maximise prey encounter rates, the detailed interactions between predators and their prey are elusive in the marine environment (Thaxter et al., 2013). Also the behavioural response to changes of both habitat quality and characteristics due to human activities are difficult to observe. It is vital that we gain an understanding of how anthropogenic impacts on the marine environment influence diving species foraging efficiency and, subsequently, how this leads to impacts of population demography, population persistence and species’ distributions.

Among marine top predators, for diving seabirds it is a major challenge to examine movements, search strategies, predator–prey interactions, and how they relate to the surrounding habitat due to the fact that they use the water column only for foraging and for a limited amount of time (Elliott et al., 2008; Doniol-Valcroze et al., 2011). Being air-breathing vertebrates, seabirds need to go back to the surface after a given diving time. Their physiological and morphological adaptations are a response to the constraints of moving in a liquid environment and diving with a limited store of oxygen (Monaghan et al., 1994; Butler & Jones, 1997; Cook et al., 2008). Under these limitations individuals try to maximise their foraging efficiency. Shape, maximum depth and duration of dives as well as recovery periods on the surface can be different among species, meaning that each species can allocate its time in different ways depending on the foraging behaviour (Schreer, Kovacs & O’Hara Hines, 2001; Tremblay et al., 2003; Elliott et al., 2008; Wilson, Quintana & Hobson, 2012).

Notably, because the cost of buoyancy changes with depth (Lovvorn & Jones, 1991; Wilson et al., 1992; Lovvorn et al., 2001; Quintana, Wilson & Yorio, 2007; Wilson, Quintana & Hobson, 2012), a diving seabird naturally goes through a vertical “heterogeneous landscape” that leads to different costs of movement during its diving cycle. To optimise the efficiency of movement or food acquisition during the foraging activity, the animal receives information about the surrounding habitat and makes movement decisions. Marine prey resources are variable in space and time and are a reflection of the interactions between ocean currents, bathymetry and other physical and biological processes (Embling et al., 2012; Scott et al., 2010; Hamer et al., 2009). Holling (1959) studied the functional responses between predators’ foraging success and prey density. Seabirds’ foraging success increases with prey density, performing a hyperbolic shaped curve similar to the type II curve of Holling’s model (Wood & Hand, 1985; Draulans, 1987; Ulenaers, van Vessem & Dhondt, 1992). At high prey densities, the benefit gained by the predator might depend on its ability to handle and digest prey. At low prey densities the predator can spend a longer time and more energy to find and capture its prey (Enstipp, Grémillet & Jones, 2007).

In the last decade the growing awareness of potential climate change impacts and the rapid depletion of fossil fuel reserves led to an increased interest in renewable sources (Rourke, Boyle & Reynolds, 2010; Kadiri et al., 2012). In particular, tidal energy is considered a promising form of renewable source, as it is an abundant and predictable supply (Kadiri et al., 2012; IEA, 2012). The arrangement of arrays of tidal devices is a developing area of research; the size and structure of the devices depend both on the specific device and the location under consideration (Shields et al., 2009). As sources of disturbance caused by human activities, their presence in the water can lead to alterations in ecosystem functions, a bottom-up trophic effect, and changes in food availability (Gill & Kimber, 2005; Gill, 2005). The indirect impacts on the structure of the communities in space and time can affect higher trophic levels as well as recruitment and distributions of marine populations. More direct effects are that fish can be attracted by any physical anomaly and a tidal energy device can be responsible for the aggregation and attraction of fish schools around the structures (Gill & Kimber, 2005; Girard, Benhamou & Dragon, 2004). Moreover, the possible overlap between the areas for the development of tidal devices and the foraging areas of top marine predators can have potentially substantial ecological impacts (Scott et al., 2014).

Most diving seabirds reach depths at which moving parts of tidal turbines can be located (Langton, Davies & Scott, 2011) and very few data are currently available concerning changes in behaviour through avoidance of the devices, changes in prey distributions and habitat characteristics. This current lack of data for making quantitative predictions and lack of general foraging theory that can inform the development of models motivated us to develop a general model about diving predators foraging underwater. Our aim is to gain theoretical understanding of the foraging efficiency of diving predators characterised by different foraging strategies in complex marine landscapes (hereafter seascape). In this study, we seek to develop some generic theory to provide insights on how diving predators such as seabirds, with different foraging strategies, are likely to be impacted by the presence of tidal turbines in the water column as source of disturbance and habitat modification.

## Methods

The model represents a seabird performing a dive cycle in a vertical cross section of the water column. The duration of the underwater search for prey is restricted due to the predator’s limited air supply. We conducted a set of simulations to evaluate the efficiency of a predator foraging in an environment affected by spatial disturbance caused by the presence of abstracted tidal devices, which could also impact the prey distribution. The model was implemented in the R language (R Development Core Team, 2013).

### The seascape

**Figure 1:**
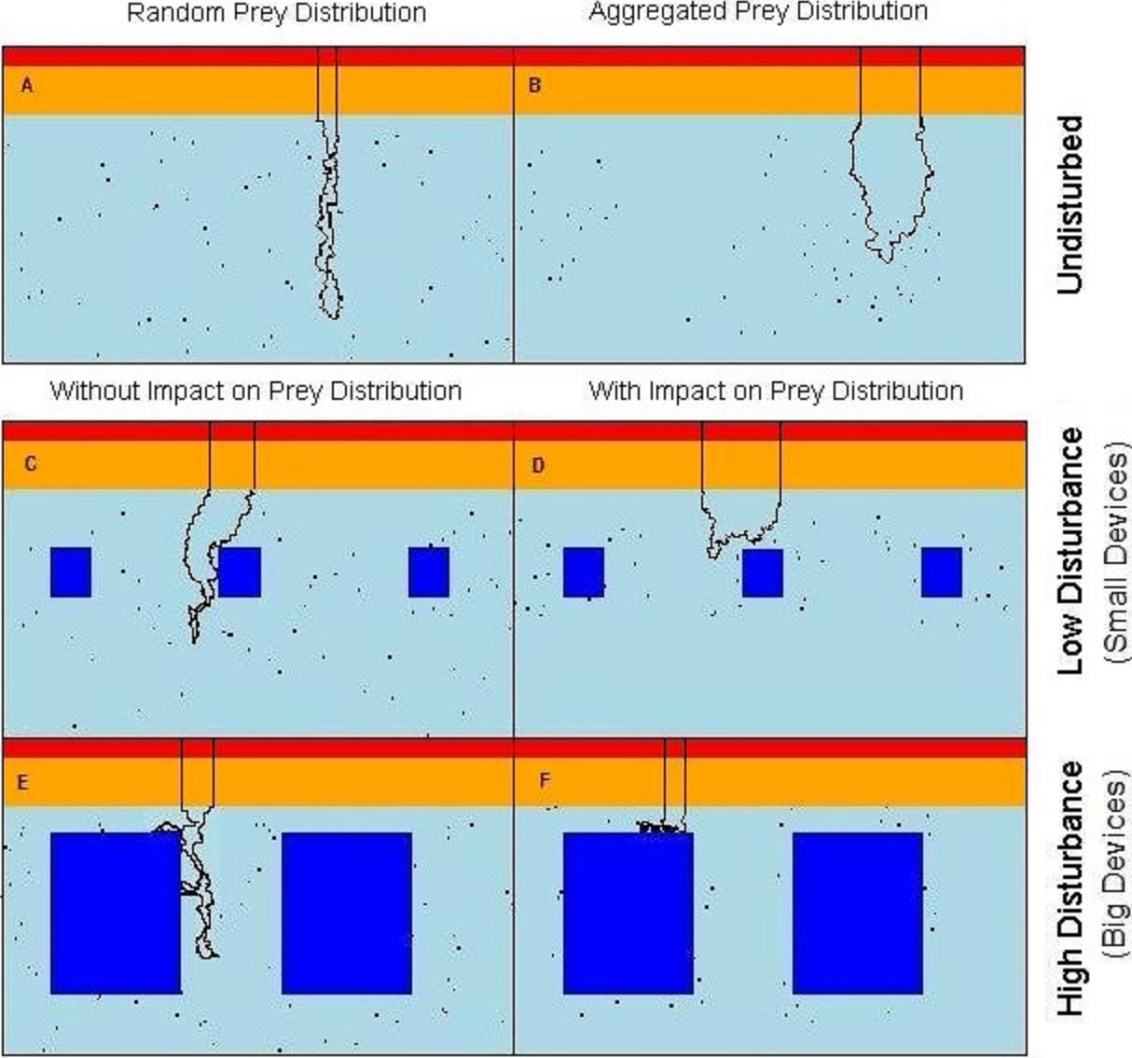
Virtual seascapes built in the model representing a cross section of the water column. In red on the top of the seascapes: high cost section, orange: medium cost section, cyan: low cost section, objects: dark blue in the low cost section, black dots: prey, black line: example of the predator movement. (A) undisturbed seascapes with random prey distribution; (B) undisturbed seascapes with aggregated prey distribution; (C) seascapes with low level of disturbance and random prey distribution; (D) seascapes with low level of disturbance spatial aggregation of prey around the devices; (E) seascapes with high level of disturbance and random prey distribution; (F) seascapes with high level of disturbance and spatial aggregation of prey around the devices.

The seascape was representing a 2-dimensional cross-section of the water column and was a matrix of 100 × 700 cells (depth × horizontal dimension, representing approximately 60 m × 420 m). The energetic costs to counteract the buoyancy decrease with depth as the increase in pressure leads to the compression of the seabirds lungs (Butler & Jones, 1997; Wilson et al., 2011). Therefore, in our model, cost of movement, being the energy that a diving predator has to expend, was an increasing function of water depth (*d*). Here, for simplicity, we took it to be a step function yielding three values of high, medium, and low cost for depths in ranges 0 ≤ *d* < 2 m, 2 ≤ *d* < 12 m, *d* ≥ 12 m, respectively (Fig. 1A).

### Disturbance

To simulate the presence of the tidal devices, impenetrable rectangular regions have been added to the deepest (low-cost) area of the water column (Figs. 1C–1F). These areas formed a barrier both for predator movement and perception. In order to observe the effect of different disturbance levels, we varied the size of the objects from either ‘small’ or ‘large’. The relative size of the small and large devices and the distance between them were realistic with regard to the size of the seabirds. In the ‘low disturbance’ scenario, 10 small objects of size 15 × 15 cells (≈ 9 × 9 m) were placed, comprising a 3.2% of coverage (Figs. 1E and 1F). The ‘high disturbance’ was achieved by placing 10 large objects of size 40 × 40 cells (≈ 24 × 24 m), comprising a 35% of coverage.

### Prey distribution

Assuming that the predators only catch and handle prey in those parts of the water column where the effort to counteract the buoyancy is reduced, available prey items were distributed only in the low cost section of the seascapes (Fig. 1A). Each prey item occupied a single matrix cell (and only one prey per cell was allowed) and was assigned a relative “benefit” value *B* = 1,000 energetic units. The number of prey items was fixed (*n* = 700, 1% of the total cells) in all scenarios, but the presence of the objects in the water could affect the distribution and spatial density of prey.

Two undisturbed (i.e., without devices) vertical seascapes were simulated, one where all prey items were each distributed randomly with equal probability anywhere within the low cost section so to simulate completely randomly spaced and dispersed prey items. The second scenario without devices has the prey items aggregated in randomly distributed clusters of random size (keeping a total of 700 prey, see R code in the Data S1 for more details). The contrasting outputs from these two scenarios allow the quantification of the difference between completely random prey and aggregated prey patches, the more likely normal situation (Fréon et al., 2005).

The presence of objects slightly affected the overall local density of prey, as they effectively concentrate the 700 individuals into a smaller area (Fig. 1). The average prey density comprised 0.034, 0.035, 0.037 fish/m^2^ of the searching area in the none, low- (small devices), and high-disturbance (large devices) scenarios without attraction to the devices, respectively. Note that we have also run simulations where we controlled for density (rather than controlling for abundance as in the presented results) and we find the effect to be very small and certainly does not account for the different results obtained with the introduction of disturbances. (See Fig. S1 for details.)

Within the “attracting” scenarios, where the devices attract fish, the prey aggregated around each object. The probability of there being prey decreases outwards from the prey cluster centre. Locational *x* and *y* coordinates, of each *n* prey locations around each device (the same *n* for each device for all simulations), followed a bivariate normal distribution. Note that a random draw was repeated if the prey fell within the device area, so that a prey item never overlapped the devices. With the low level of disturbance the prey were aggregated around each object within an area of 1350 cells (≈ 500 m^2^) with a local prey density of 0.139 fish/m^2^. With the high level of disturbance the prey were aggregated around each object within an area of 1764 cells (≈ 650 m^2^) with a prey density of 0.108 fish/m^2^ (Figs. 1B–1F) (see R code in the Data S1 for more details).

### Predator’s searching behaviour

The search for prey started once the predator reached the ‘low cost’ section of the water column (Fig. 1), and was limited only to the low cost section, as we assumed that the movement costs (i.e., counteracting the buoyancy) in the remaining sections were too high to be advantageous for the predator to search for prey there (Lovvorn et al., 2004; Morales & Ellner, 2002). The predator perceptual range was 2 units (1.2 m) around the predator position. If a prey item fell within this range, the predator directed toward the prey. Otherwise, movement decision was affected by the eight neighbouring cells. The movement direction was affected by both the directional persistence, and a drive to go downwards. Trajectories generated in this way resembled the shape of general diving profiles performed by diving seabirds as shown in Lescroël & Bost (2005). Next position was one of the eight cells adjoining the current position, selected randomly with relative preference value *λ*. If the selected cell fell outside the landscape or in the device region, the draw was repeated until it fell within an unoccupied cell.

The preference *λ*_*x*,*y*_ for cell at relative coordinates *x* and *y*, was a result of two components, the directional persistence (direction of the preceding step) and downward draw, and was calculated as:

(1) \begin{eqnarray*} {\lambda }_{x,y}=[\kappa \mathrm{CNorm}(\mathrm{diff}({\alpha }_{t-1},{\beta }_{x,y}),1)+\tau (\mathrm{diff}(\delta ,{\beta }_{x,y}))]\mathrm{dist}(x,y)^{-1} \end{eqnarray*}

where: *κ* is the strength of directional persistence; CNorm(*μ*, *σ*) is a wrapped normal probability density function, with mean direction *μ* and standard deviation *σ*; diff(*α*, *β*) represents angular difference between *α* and *β*; *α*_*t*−1_ is the movement direction of the previous step; *β*_*x*,*y*_ is the direction to cell located at *x* and *y*; and *δ* is the direction downwards (to the cell beneath current position, so *δ* = *β*_0,1_). The strength of the directional persistence, *κ*, is a function of the number of steps since the last prey encounter, *m*, defined as *κ* = min(*κ_min_* + log(*m*), *κ_max_*), where *κ* is bound between *κ_min_* = 0.5 and *κ_max_* = 3.0, so the resulting movement followed an area-concentrated search (Fauchald & Tveraa, 2003). *τ*(*α*) is a strength of the downward draw, and yields 1 when *α* is 0, and 0 otherwise. To adjust for the square grid, sum of the two components was weighted by distance to the cell center (1/dist(*x*, *y*)).

### Predator’s complete diving cycle

We simulated single dive cycles, in which the trajectory started and ended at the surface and was divided into 3 phases: descent, search and ascent. We assumed that during descent, seabirds might have to stroke or paddle continuously in order to maintain speed against profile drag and buoyancy (Lovvorn, Croll & Liggins, 1999) so the descent lasted until the predator reached the low cost section of the seascape (Fig. 1A, black line). Next, assuming that during the search seabirds alternated gliding with bouts of stroking/paddling while swimming (Watanuki et al., 2003), the predator moved in search of prey until it captured *N_prey_* (see below) or reached the maximum duration of the searching phase, whichever occurred first, and subsequently it returned to the surface.

At each step, the predator expended an amount of energy depending on the depth it was in (see above) and the diving phase. During the descending and searching phases the per step energy expenditure corresponded to the movement cost *C_s_* associated with each section *s* of the seascapes. During the ascending phase, because of the opposite effect of the hydrostatic pressure, the per step energy expenditure corresponded to a constant cost *C_asc_*. If the predator encountered a prey (i.e., if prey was in the same cell as the predator), it gained *B* energy units, and that prey was removed. During the ascending phase the predator moved directly to the surface. Movement speed was constant in all phases, one unit (≈ 0.6 m) per time step.

We examined three strategies differing in the maximum number of prey items that the predator was able to catch during the searching phase: one, three, and unlimited (denoted as *P*_1_, *P*_3_ and *P_N_* respectively, Table 1).

**Table 1:** Summary of the model parameters used for the simulations. See Data S1 fo more details.

Parameters used in the simulations	Values (s)
Seascape size (unit = cells)	100 × 700
Total number of food items in the seascape	700
Number of clusters simulated	30
Number of food items per cluster	Random selected from 1 to 50, with total = 700
Foraging strategies simulated: *P_N_*, *P*_3_, *P*_1_	*P_N_*: able to catch unlimited prey items
	*P*_3_: able to catch a maximum of 3 prey items
	*P*_1_: able to catch a maximum of 1 prey items
Energy content of the prey item	1,000
Number of devices simulated	10
Sequence of searching time simulated (unit = time step)	From 10 to 300 every 10
Maximum dive time sequence simulated (unit = time step)	From 210 to 500 every 10

### Simulations

The simulations were run in a full factorial design of the three disturbance levels (none, small and large devices), and two types of prey distribution: uniform and aggregated, with aggregation specific to devices when they were present. For each combination of parameter values (summarized in Table 1) we ran 500 replicates of an individual dive cycle. The starting point of each dive was a randomly selected cell of the surface (top row of the landscape grid). Prey locations were different in each run, meaning that in both uniform and aggregated distribution the location of the prey was randomly selected respectively within the whole foraging area and within the area chosen for the aggregation.

In order to estimate the impact of the devices on the efficiency of the searching strategy, we calculated the time taken to find first prey, as well as prey encounter intervals, up to 3rd prey. For this purpose we used trajectories of the searching phase only (i.e., within the lowest part of the water column).

Then, to assess the effect of the devices on different foraging strategies, we introduced a full diving cycle, including the descent and ascent phase. In the six environmental conditions described above, three types of predators were tested (*P*_1_, *P*_3_, *P_N_*), with 20 different Maximum Dive Durations allowed (*MDT*, ranging from 218 to 408 time steps to represent the range of dive durations across multiple species). The predator’s vertical position (*y*) at each time step, and the number of prey encountered (*N_prey_*) was recorded.

For each combination of parameters, predator’s foraging success was estimated. The foraging trip was considered successful when the predator caught at least 1 prey. Foraging efficiency index was calculated as the ratio of the benefit gained in a diving cycle, and the total energy expended during the dive cycle:

(2) \begin{eqnarray*} \varphi ={N}_{p r e y}b/\sum c(y) \end{eqnarray*}

where *N_prey_* is the number of prey caught during the dive cycle, *b* is the benefit of a single prey, and the last term is the total expended energy, being a sum of the energy spent in making each step of the dive cycle of *m* steps. *c* is the function of movement cost (see above) at depth *y_i_*, at which the predator was present at step *i*.

The mean foraging efficiency for each predator strategy (*P_N_*, *P*_1_, and *P*_3_) was plotted against the maximum dive time. This was done to observe how the foraging efficiency was related to varying maximum dive times.

## Results and Discussion

Particularly in the marine environment, the circumstances surrounding prey capture are largely unknown. Vertical movements (diving) of top marine predators are considered one of the 4 phases that characterise the foraging behaviour of diving marine predators: vertical movement (diving), horizontal movement, habitat use, and resultant prey capture (Austin et al., 2006). In this model we started to develop and test the foraging events only occurring during this phase. We focused on the vertical movements in order to understand at a finer scale the dynamics of a diving predator encountering its prey and the effects of different prey distributions and habitat heterogeneity characteristics.

When foraging in the seascapes, all simulated predators experienced the same physiological constraints and had access to the same information from the surrounding habitat. Despite this, distinct movement patterns emerged in different seascapes highlighting the effect of the prey distribution and the presence of the devices (Fig. 1).

### Searching efficiency

**Figure 2:**
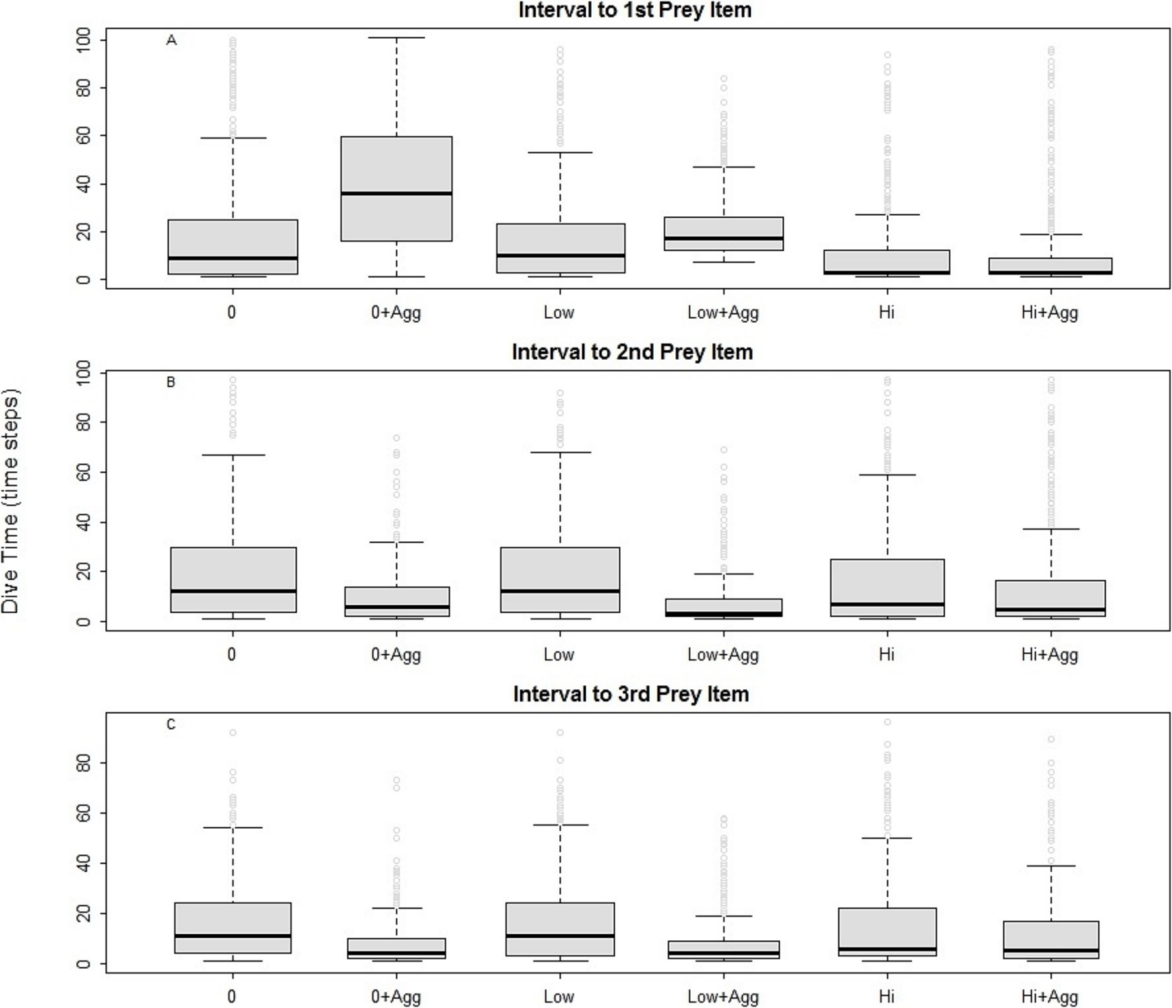
Intervals between prey catches in a general predator. 0, Undisturbed seascapes; 0 + Agg, Undisturbed seascapes with aggregated prey distribution; Low, Low disturbance without impact on the prey distribution; Low + Agg, Low disturbance with impact on the prey distribution; Hi, High disturbance without impact on the prey distribution; Hi + Agg, High disturbance with impact on the prey distribution.

For all the predators simulated, the searching efficiency depended on both the distribution of the prey and the device encounter rate (Fig. 2). Both low and high disturbance affected the prey encounter intervals. In general, locating the first prey item was the most time consuming in all examined landscapes except in those characterised by high disturbance. Searching was most efficient where prey were aggregated (Fig. 2, 0 + Agg, Low + Agg, Hi + Agg). In the undisturbed seascape (Fig. 2, 0 + Agg), the longer time needed to find the first prey item corresponded to the time taken to find the first prey cluster. Once a cluster was found, the probability of finding subsequent prey was higher due to spatial autocorrelation of the prey and hence increased local prey density. The spatial aggregation of prey around small devices led to a higher prey encounter rate compared with the other scenarios (Fig. 2, Low + Agg).

The presence of high disturbance (Fig. 2, Hi, Hi + Agg), allowed the predator to find its first prey sooner due to the higher local density of the prey and the higher possibility for the predator to encounter the device and change its movement direction. At the same time, this scenario inhibited its ability to find the subsequent prey, especially when the prey was randomly distributed (Fig. 2, Hi). This suggests it is primarily the prey aggregation that is influencing the search success (Bartoń & Hovestadt, 2013), followed by the type of disturbance and its impact on the prey distribution.

### Habitat heterogeneity vs. foraging strategies

**Figure 3:**
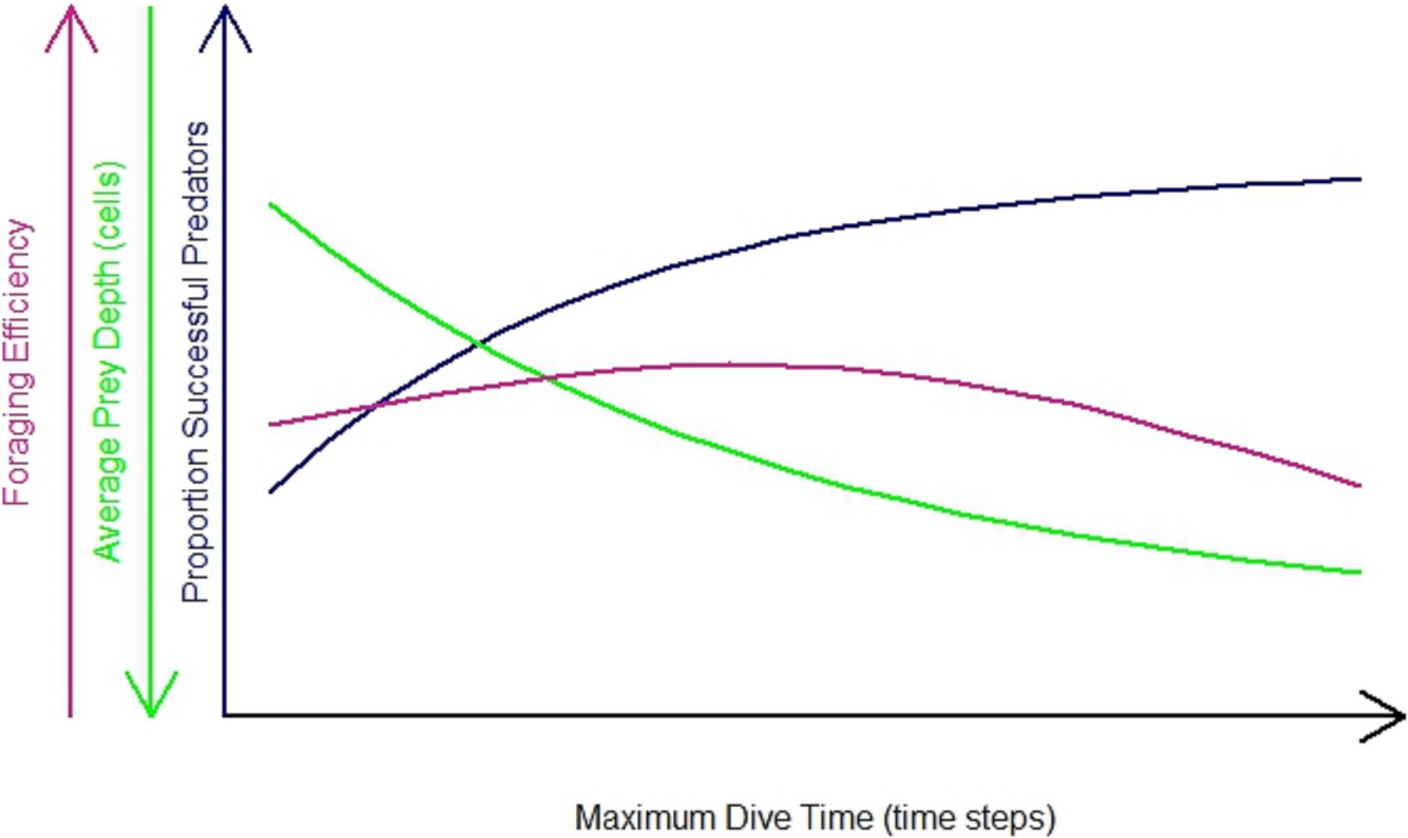
General predator in the undisturbed seascapes with random prey distribution. Proportion of successful predators per maximum dive time (blue line), foraging efficiency (purple line) and average prey depth (average depth where prey were caught) (green line).

The impact of the differences in habitat heterogeneity on the foraging efficiency of the diving predators depended on their foraging strategy (‘n-prey loader’), on the searching efficiency and on the time spent underwater. In general, the increase in Maximum Dive Time (MDT) resulted in an increase in the foraging success of the predator (Fig. 3, blue line). During longer dives, the longer searching time available gave the opportunity to the predator to cover a greater diving distance, increasing the probability for the predator to succeed during the foraging event. At the same time, the combination of the directional persistence and the downward draw led the predator to dive deeper. This increased the probability for the predator to catch prey deeper in the seascapes (Fig. 3, green line), but it caused a longer ascending phase. So the success of the foraging event, the average depth where the predator caught the prey and the length of both searching and ascending phase contributed to the variation of the foraging efficiency. After reaching a maximum threshold, the resulted decrease of the foraging efficiency was due to the predator spending more time underwater and catching its prey mainly in the deeper part of the seascapes (Fig. 3, purple line). The foraging efficiency was assessed for each of the 3 foraging strategies (*P*_1_, *P*_3_, *P_N_*) and for different dive times (Table 1) and was expressed as the benefit to cost ratio (number of prey caught per energy expended, see ‘Methods’ for details).

### Foraging pattern *P_N_* (multiple loader)

**Figure 4:**
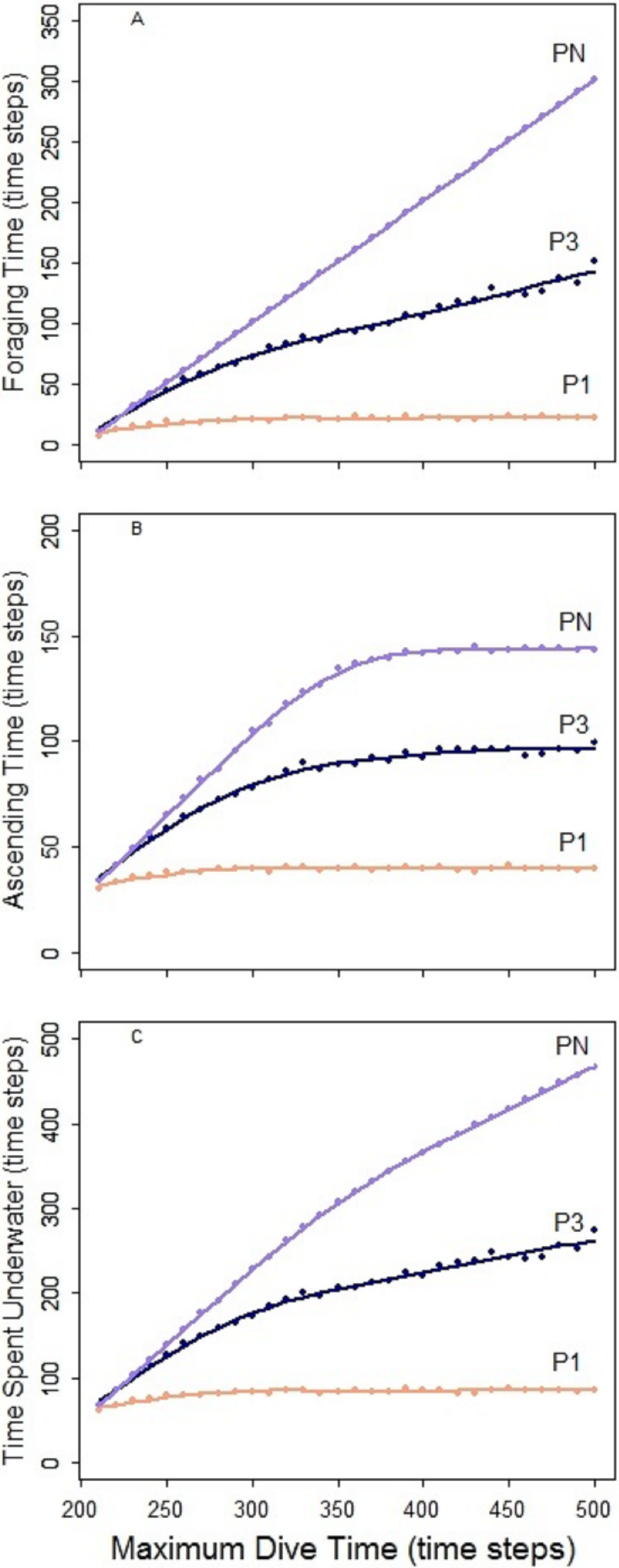
Predator *P_N_*, *P*_3_ and *P*_1_. Foraging time (A), ascending time (b) and time spent underwater (C).

**Figure 5:**
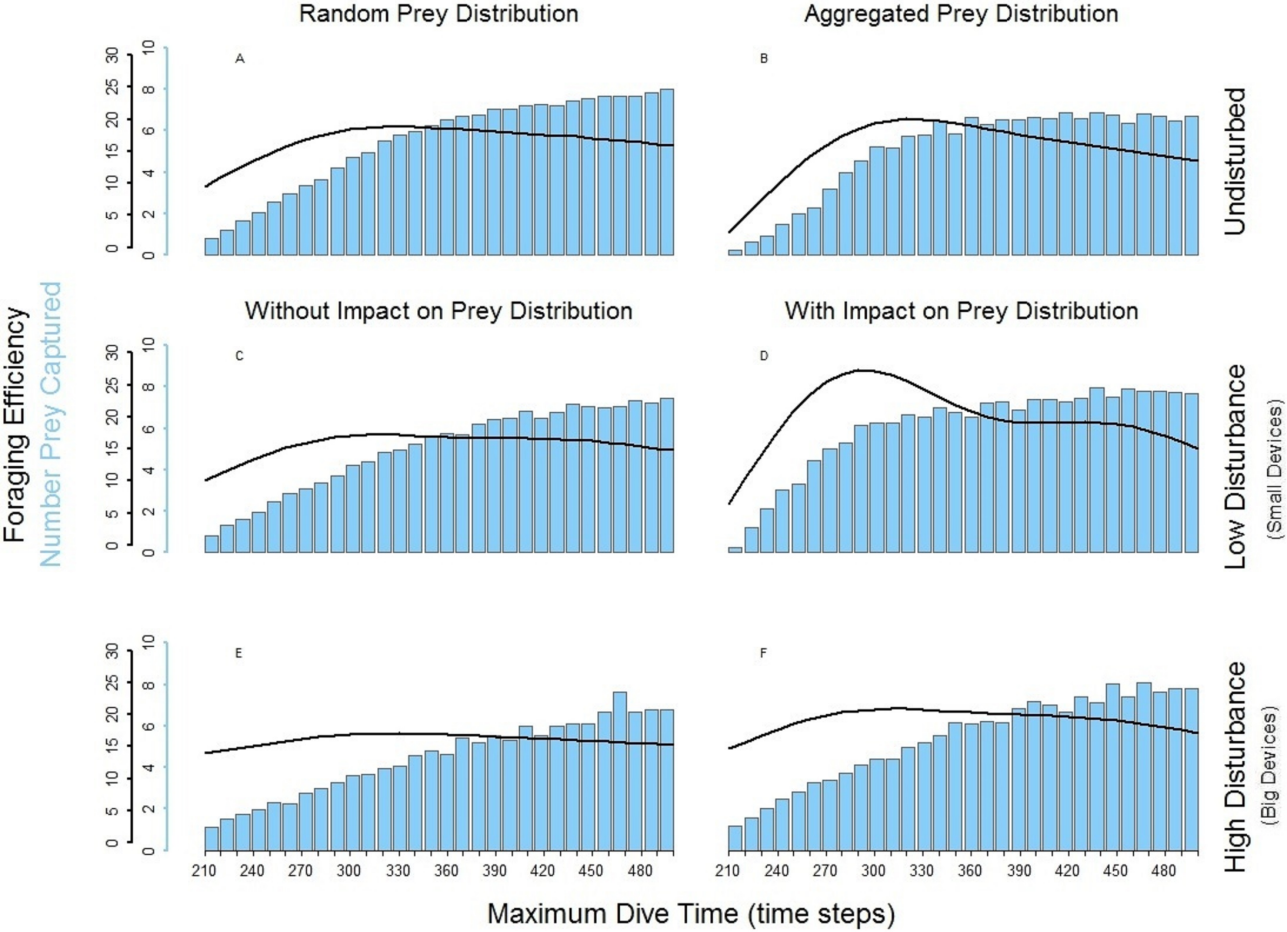
Foraging efficiency (black line), and Number of Prey Captured (bar plot) of the multiple loader *P_N_* among different dive times in response of the low and high level of disturbance. (A) basic seascapes and random prey distribution, (B) basic seascapes and aggregated prey distribution, (C) low level of disturbance and random prey distribution; (D) low level of disturbance and spatial aggregation of prey around the devices; (E) high level of disturbance and random prey distribution; (F) low level of disturbance and spatial aggregation of prey around the devices.

In the foraging strategy of the predator *P_N_*, the number of prey caught was limited only by the maximum dive time. It is the only considered strategy where the maximum time was always equal to the effective/actual dive time. In all scenarios, longer searching time for the prey allowed *P_N_* to capture an increasing number of prey (Fig. 5, bar plots). Despite this, the increasing time spent underwater (Fig. 4C, *P_N_*) led to a decrease of the foraging efficiency after a given MDT. Both low and high disturbance affected the pattern of the foraging efficiency depending on the effect on the prey distribution and these effects are explained separately next.

The spatial aggregation of the prey created a valuable area for successful foraging activity (Bartoń & Hovestadt, 2013). In the undisturbed seascape with aggregated prey distribution (Fig. 5B) *P_N_* could get the advantage of catching a larger number of prey once it found the prey cluster. Despite this, the longer time spent underwater and the possibility that *P_N_* could not detect other clusters within its perceptual range negatively affected the overall foraging efficiency of the whole dive cycle. The aggregation of the resources around small devices forced *P_N_* to stay mainly in the upper part of the seascapes. Performing a shorter diving cycle and spending less energy in its ascending phase, *P_N_* achieved a higher foraging efficiency (Fig. 5D). With a longer MDT, *P_N_* could also reach the deeper part of the water column. So during both search and ascent, it could encounter the devices and spend more time and energy, which negatively affected the foraging efficiency (Fig. 5D). The foraging efficiency of *P_N_* was positively affected by the presence of high disturbance, especially in short dives (Figs. 5E and 5F).

### Foraging pattern *P*_1_ (single loader) and *P*_3_ (triple loader)

The foraging strategies *P*_3_ and *P*_1_ involved the limit for number of prey items per single dive cycle. Different to *P_N_*, the time necessary to catch the required number of prey was shorter than the MDT allowed within the simulation. Consequently, increase of MDT beyond the point when the prey capture limit was reached had no effect on the dive nor its efficiency that levels off at certain MDT (Fig. 4). Shorter foraging and ascending phases, resulted in consequently shorter time spent underwater (Figs. 4A–4C). Highest foraging efficiency of both *P*_3_ and *P*_1_ occurred when prey was distributed randomly.

**Figure 6:**
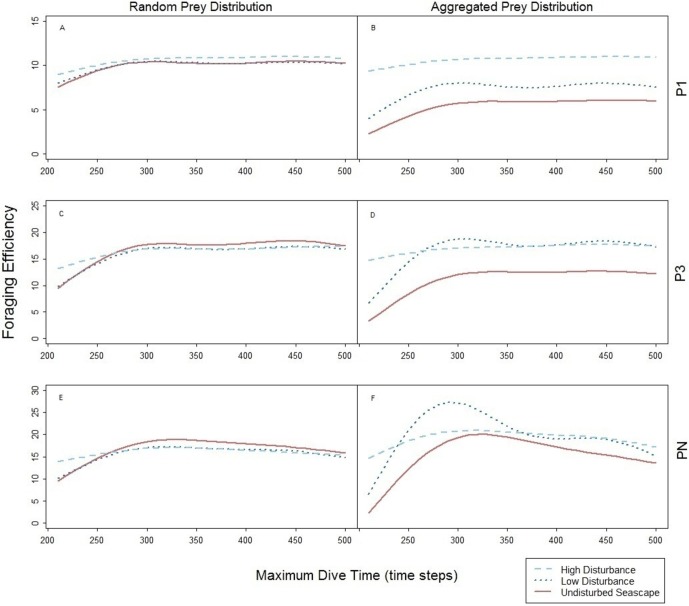
Foraging efficiency of the three predators *P*_1_ (A and B), *P*_3_ (C and D), and *P_N_* (E and F) among different maximum dive times. Undisturbed seascape (red solid line) and response in presence of the low (dotted blue line) and high level (dashed cyan line) of disturbance.

The spatial aggregation of prey in the undisturbed seascape (Figs. 6B–6D) did not represent an advantage for both *P*_3_ and *P*_1_, due to the ‘loading limitation’, unlike in *P_N_* (Fig. 6F). Their foraging efficiency was indeed lower with aggregated than with random prey distribution (Figs. 6A–6D, dotted line). However, relative to the foraging efficiency in the undisturbed landscape the increase of the local prey density when prey aggregated around the small objects was beneficial especially for *P*_3_ (Fig. 6D, dotted line).

The spatial aggregation of the prey around the big devices combined with device size allowed both *P*_3_ and *P*_1_ to reach a similar higher level of foraging efficiencies obtained with random prey distribution (Figs. 6B and 6D, dashed line). However in *P*_1_, foraging efficiency is strongly affected by the time to find the first prey item (the only one needed) which can occur sooner in the presence of both large devices and subsequently higher local prey density (Figs. 6A and 6B).

### Comparisons with species specific behaviours

Quantification of prey capture rates and direct observations of feeding events is challenging for diving seabirds that catch their prey underwater. Underwater behaviour and prey capture rates have been observed only in few seabird species using animal borne video camera (Poganis et al., 2000; Takahashi et al., 2004; Watanuki et al., 2008), stationary underwater video cameras (Crook & Davoren, 2014) and data storage loggers that record prey ingestion (Enstipp et al., 2006; Watanabe & Takahashi, 2013). Moreover, bioenergetics models have been developed to estimate also how prey capture rates vary with different prey availability, prey distribution, prey size and patch quality (Grémillet et al., 2003; Enstipp, Grémillet & Jones, 2007; Harding et al., 2009; Thaxter et al., 2013). Our study considers how an increasing complexity in a foraging environment can affect the time required to catch prey underwater and as consequence the foraging efficiency. As shown in our model, a simple change in prey distribution can affect the predator’s searching time necessary to catch a set amount of prey items, affecting its foraging efficiency (Figs. 6A and 6B). Seabirds such as the Little auk (*Alle alle*) feed mainly on copepods (Harding et al., 2009) and can be considered similar to the multiple loader *P_N_* simulated in this model. The zooplankton community composition is closely linked to oceanographic conditions, and the availability of the different species will directly affect the number of prey items that the species will need to consume to balance their energy budget (Piatt & Harding, 2007; Harding et al., 2009).

Common guillemots (*Uria aalge*) can be considered close to a single prey loader or triple prey loaders in terms of prey capture rates per dive (Thaxter et al., 2013). Needing only a limited number of prey (from 1 to 3) , the predators *P*_1_ and *P*_3_ do not gain an advantage in locating a prey patch as the multiple prey loader does. Our current model does not simulate prey response to the presence of the predator, however, the fact that Common guillemots might prefer solitary prey or prey in low density schools, as suggested in Crook & Davoren (2014), might be due to the combination of confusion effect, i.e., the difficulty in attacking a prey within a fish school and their foraging strategy.

It has been shown that seabirds might show different foraging strategies while performing self-feeding and chick provisioning dives (Ydenberg et al., 1994; Davoren & Burger, 1999). Individual predators might therefore be affected in different ways, depending on their current foraging strategy.

### Future research directions

Species respond differently to fluctuations in the composition and availability of prey depending on their ecology, physiology, and life history traits. Data on the predator response to different prey distribution and composition is also limited by the potential variability of the response both at an individual and species level and requires an understanding of the functional relationship between each prey preference and availability (Einoder, 2009). The flexibility of the foraging strategies, in terms of prey preference, is a factor that might affect the foraging efficiency of these diving predators, increasing the complexity of a scenario where the presence of a increased heterogeneity might change the density, the distribution and the composition of each prey type. In response to poor food quality Common guillemots are able to change both the type of prey caught and increase the amount of time spent foraging, expressed as time spent in the whole foraging trip (Uttley et al., 1994; Barret, 2002; Harding et al., 2007). As single prey loaders as the predator *P*_1_, their foraging efficiency might then be particularly affected by the energy content of the food items (Wanless et al., 2005). These aspects, as well as the possibility to associate single dives to a foraging bout and multiple foraging bouts to a foraging trip were not implemented in the current model but they need to be considered in future developments.

Seabirds can possibly make use of experience, memory and local enhancement when foraging (Irons, 1998; Davoren, Montevecchi & Anderson, 2003). They can associate the surroundings of devices with more profitable places to forage, increasing their foraging success (Grünbaum & Veit, 2003) and number of prey located and captured in these areas (Nevitt, Losekoot & Weimerskirch, 2008). Tidal turbines and their support structures have been shown to have the potential to act as fish aggregation devices (Viehman & Zydlewski, 2014) as have other anthropogenic structures such as the foundations of offshore wave power devices (Langhamer & Wilhelmsson, 2009), offshore wind farms (Willhelmsson, Malm & Öhman, 2006), and decommissioned oil platforms (Soldal et al., 2002). Large devices may be more visible from longer distances, perhaps also from water surface, which can further decrease potential search area and improve search success. It was beyond the scope of our current simple model to implement such complex behaviours, but such behaviours should be considered when assessing the impact of tidal turbines on diving predators foraging success. In order to fully understand the effect that the increase in heterogeneity from renewable tidal devices can have on seabirds’ foraging characteristics and the vulnerability of the species, the potential alterations on the foraging areas and the prey distributions need to be taken into account (Furness & Wade, 2012). This model represents a valuable starting point for major modelling explorations concerning energetic budgets during foraging tasks, how animals deal with environmental uncertainties and complexity during their search behaviour and how their foraging efficiency is likely to be affected (Bartoń & Hovestadt, 2013). Behavioural necessities of a diving seabird such as limitations of diving depth and the spatial distribution of resources can affect the spatial interaction between predators and prey and hence the foraging efficiency (Sih, 1998; Fauchald, 2009).

Identifying factors that lead to successful foraging in predators is important (Austin et al., 2006) and the combination of empirical data and new modelling protocol will lead to both a better understanding of the mechanistic factors associated with successful foraging. Future modelling will be able to take advantage of understanding gained from data from real animal movements from tagging data in order to develop a more refined strategic understanding of how diving predators of different foraging behaviours are likely to be impacted by distrubances. Additionally, details on animal movements available with the GPS technology, telemetry data, and data storage tags, allow quantification of modes of movements (i.e., Morales et al., 2005; Miramontes, Boyer & Bartumeus, 2012; Regular, Hedd & Montevecchi, 2013) will facilitate the development of parameter rich models capable of making quantitative predictions of how particular species are likely to respond to the introduction of disturbances in particular environments. We believe that there is a need for the dual development of strategic models (such as that we have presented) and tactical models in this area. It will be important that both begin to incorporate increasing complexity. One particularly important consideration is increasing the spatial and temporal extent of the model such that birds can undertake many more dives over a period of days, are able to move between different foraging areas and are able to base the decisions that they make on memory of past foraging success. Also, understanding motivations of movements and how different observed patterns may depend upon the distribution of resources and other species in the seascapes are key factors in order to clarify the predator–prey interaction in complex and variable habitats.

Importantly, we emphasise the need for development of 3-Dimensional models, involving more complex and realistic searching strategies. Such a development will be somewhat more challenging and computationally expensive but it can provide further insights for the development of new theoretical models, providing a better understanding of the mechanisms and consequences of animal movements in 3 dimensions under different habitat and prey availability scenarios and enabling exploration of predator foraging theory and predator response to environmental uncertainties and spatial heterogeneity caused by human activities. Additionally, by developing 3-D models it will be more straightforward to directly link models to tagging oriented data and begin the challenging task of moving from strategic theoretical models to tactical species based models directly useful for application.

## Conclusions

Because of the natural variability of the marine resources and both physiological and morphological adaptations of diving seabirds, different seabird species perform different foraging strategies in order to maximise their foraging efficiency. Due to the difficulty in observing foraging birds and prey behaviours simultaneously there is a current lack of data on the possible relationships between prey abundance, spatial distribution and predator foraging efficiency. The future addition of large developments of man-made structures to the marine environment increases the need to ask detailed ecological questions about how these predators behave and what their reactions may be to a changing level of heterogeneity in the environment. The theoretical foraging model presented in this work provides an important tool to begin to explore predator responses to spatial heterogeneity and differences in prey behaviour. The results of this initial 2 dimensional model suggests that the introduction of increased heterogeneity via man-made structures such as tidal turbines will have differing effects on the foraging efficiency of species with different foraging strategies (i.e., single vs multiple loaders). Foraging efficiencies and foraging behaviours will also be influenced by the reaction of prey to the level of heterogeneity suggesting changes in search behaviour and predator–prey interactions due to changes in prey behaviour around structures. This modelling framework along with new detailed movement data of diving seabirds can provide new insights to the foraging theory and directions for the implementation of the ecosystem-based management strategies in those areas where human activities are likely to have ecological impact.

## Supplemental Information

10.7717/peerj.544/supp-1 Figure S1 Simulated scenario with different prey densities Default prey density (700 prey, density = 0.034 fish/m^2^, red dashed line), prey density the same as in the seascape with high level of disturbance (733 prey, density = 0.037 fish/m^2^, purple dashed line) and scenario with a total of 1000 prey and density = 0.050 fish/m^2^ (yellow dashed line). Importantly, controlling for density does not alter the shape of the relationship. The output from the simulation with default prey density and both low and high disturbance (respectively dark blue line and cyan line) has been added to show the qualitative effect that the disturbances have on the shape of the relationship Foraging Efficiency — Maximum Dive Time.

10.7717/peerj.544/supp-2 Data S1 R code used to run the model and generate the raw data used in the analyses
