# Supplementary figures and images for: Modelling foraging movements of diving predators: a theoretical study exploring the effect of heterogeneous landscapes on foraging efficiency
Source: PeerJ. 2014 Sep 11;2:e544. doi: 10.7717/peerj.544 (PMC4168760; doi:10.7717/peerj.544)

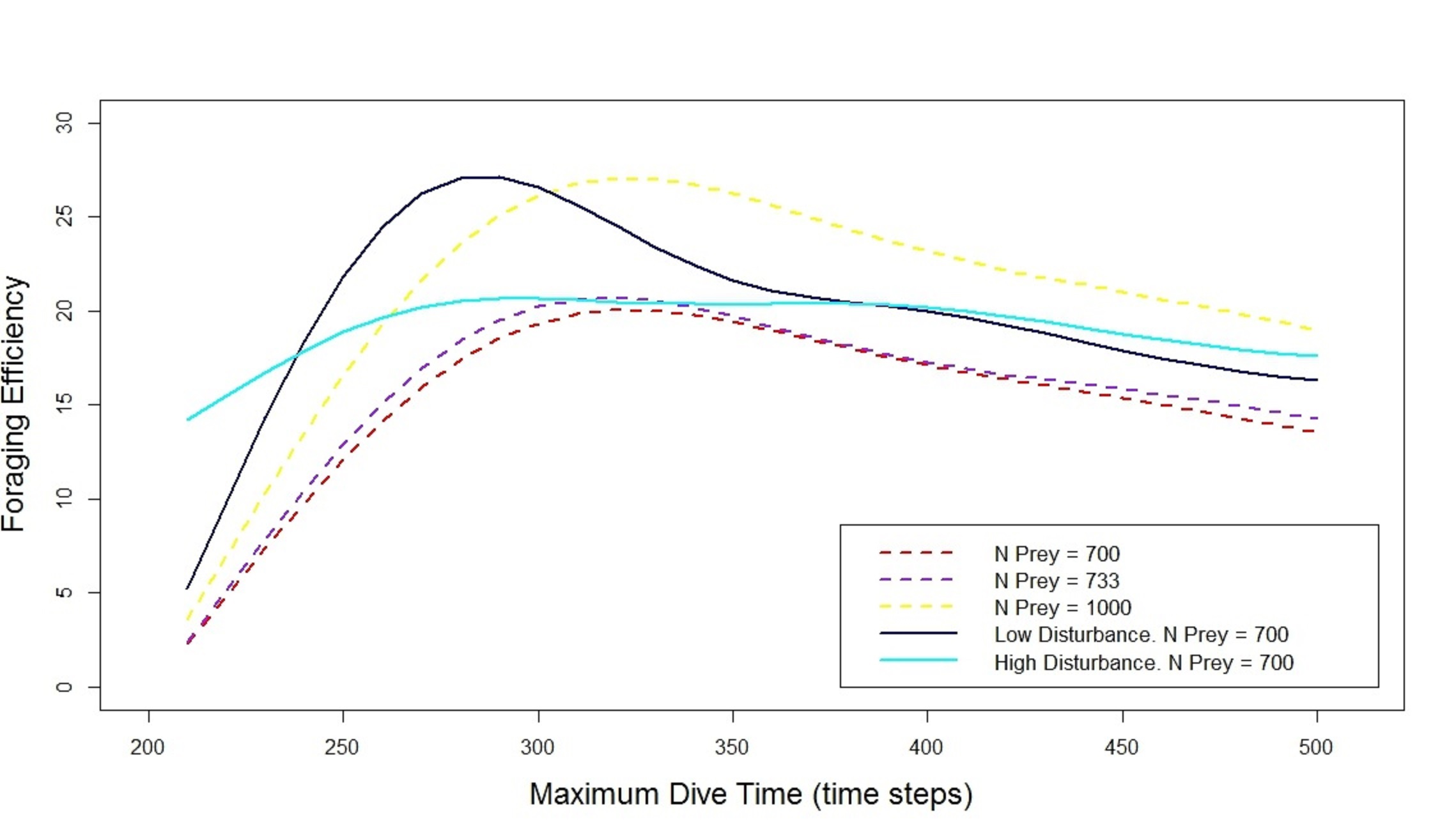

Supplement: Figure S1 — Default prey density (700 prey, density = 0.034 fish/m2, red dashed line), prey density the same as in the seascape with high level of disturbance (733 prey, density = 0.037 fish/m2, purple dashed line) and scenario with a total of 1000 prey and density = 0.050 fish/m2 (yellow dashed line). Importantly, controlling for density does not alter the shape of the relationship. The output from the simulation with default prey density and both low and high disturbance (respectively dark blue line and cyan line) has been added to show the qualitative effect that the disturbances have on the shape of the relationship Foraging Efficiency — Maximum Dive Time. [file peerj-02-544-s001.png]
